# Dynamic tilting in perovskites

**DOI:** 10.1107/S2053273322011949

**Published:** 2023-01-23

**Authors:** Christopher M. Handley, Robyn E. Ward, Colin L. Freeman, Ian M. Reaney, Derek C. Sinclair, John H. Harding

**Affiliations:** a Department of Materials Science and Engineering, University of Sheffield, Sir Robert Hadfield Building, Mappin Street, Sheffield, S1 3JD, United Kingdom; b Digital Research Service, University of Nottingham, University Park, Nottingham, NG7 2RD, United Kingdom; Czech Academy of Sciences, Czech Republic

**Keywords:** perovskites, tilt, diffraction, molecular dynamics, superlattice

## Abstract

A new computational analysis of tilt behaviour in perovskites is presented. This includes the development of a computational program – *PALAMEDES* – to extract tilt angles and the tilt phase from molecular dynamics simulations. The results are used to generate simulated selected-area electron and neutron diffraction patterns which are compared with experimental patterns for CaTiO_3_. The simulations not only reproduced all symmetrically allowed superlattice reflections associated with tilt but also showed local correlations that give rise to symmetrically forbidden reflections and the kinematic origin of diffuse scattering.

## Abstract 2

A new computational program to analyse and extract tilt data from molecular dynamics simulations of perovskites is presented and results compared with experimental data.

## 1. Introduction

The perovskite structure has the general formula *ABX*_3_ and is made up of a network of corner-sharing *BX*_6_ regular octahedra. Each set of eight connected octahedra encloses a cuboctahedron occupied by an *A* site. This cubic structure is often seen at higher temperatures but at lower temperatures structural distortions (tetragonal, orthorhombic, rhombohedral *etc*.) are observed. These are linked to their useful material properties, such as ferroelectricity (Chauhan *et al.*, 2015 ▸), piezoelectricity (Reaney, 2007 ▸) and relaxor behaviour (Garten *et al.*, 2016 ▸). Fully characterizing and controlling distortions has been a topic of interest for crystallographers for decades (Reaney, 2007 ▸; Beanland, 2011 ▸; Glazer, 1972 ▸, 1975 ▸). The best-known structural transitions involve displacement or off-centring of cations. For example, barium titanate is cubic above the Curie temperature, *T*_c_, but undergoes a series of distortions to a tetragonal, orthorhombic and finally rhombohedral lattice as the temperature decreases (Kwei *et al.*, 1993 ▸), a result of a sequential displacement of the Ti ion from the centre of the octahedron along the [001], [011] and [111] directions, respectively. The commonest type of distortion involves rotations or tilting of the octahedra. Traditional treatments assume undistorted octahedra, but the octahedral and cuboctahedral sites often become distorted away from ideal (Reaney, 2007 ▸; Glazer, 1972 ▸, 1975 ▸; Woodward, 1997 ▸). For example, in calcium titanate the Ca ion is too small to occupy the *A* sites fully, so the octahedra tilt, distorting the cuboctahedron and shortening some of the Ca—O bonds (Sasaki *et al.*, 1987 ▸). Jahn–Teller effects distort the octahedra in KCuF_3_ (Lufaso & Woodward, 2004 ▸; Okazaki & Suemune, 1961 ▸). More complex symmetries and tilting patterns occur in hybrid organic–inorganic perovskites (Amat *et al.*, 2014 ▸) and those where the *A*-site ion contains a lone pair ion such as Bi^3+^ or Pb^2+^ (Ramesh & Spaldin, 2007 ▸).

**Figure 1:**
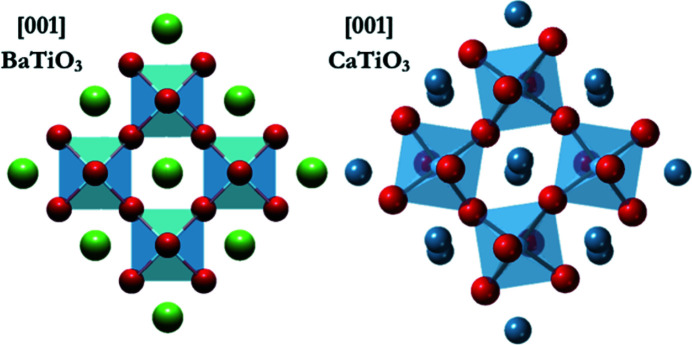
Comparison of the untilted and tilted structures of BaTiO_3_ (left) and CaTiO_3_ (right). Red, oxygen; light blue, Ti; green, Ba; dark blue, Ca ions (CrystalMaker, 2018 ▸).

**Figure 2:**
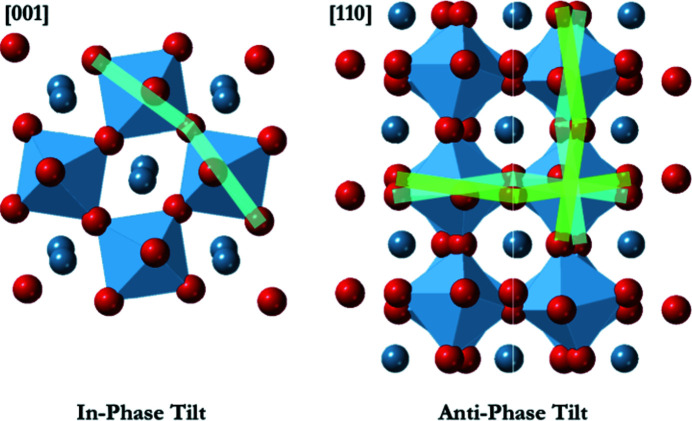
Comparison of tilting in a perovskite. Each image involves two layers of octahedra to demonstrate that each can tilt by the same magnitude, but either in the same direction or in the opposite direction, giving in-phase or anti-phase tilting, respectively.

The distortions of a perovskite structure can be rationalized using a simple ratio of ionic radii, the Goldschmidt tolerance factor, *t* (Goldschmidt, 1926 ▸):

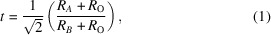

where *R_A_*, *R_B_* and *R*_O_ are ionic radii of the *A*-site, *B*-site and O-site ions, respectively. If *t* ≤ 0.97, the octahedra tilt (Reaney & Ubic, 1999 ▸) but, although tilting is absent for *t* > 0.97, other instabilities may still occur. Glazer defined the mechanisms of tilt of the octahedra, which result in a series of unique configurations (Glazer, 1972 ▸, 1975 ▸). Glazer tilting uses extrinsic rotations, *X*–*Y*–*Z*, with respect to a pseudo-cubic frame of reference. He assumed that the octahedra do not distort away from the ideal internal bond angles and bond lengths, and that all tilts are <15°. The rotation of one octahedron about an axis results in the rotation of the connected octahedra, with the shared oxygen ion along that vector acting as a hinge. The connected octahedra rotate by the same magnitude as the original octahedron, but in the opposite sense like connected cogs (Fig. 1 ▸). If we consider the twisting of the octahedra in one layer, and the rotations of the octahedra in the corresponding layers above and below, we can see that these tilts need not twist in the same sense. We can describe these tilting patterns as either in-phase (the octahedra in each layer twist in the same sense as the layers above and below) or anti-phase (the rotation is in the opposite sense) (Fig. 2 ▸). The three intrinsic rotations are termed *a*, *b* and *c*, with the in-phase and anti-phase tilts labelled + and −, respectively.

In the Glazer tilt system, the symbol *a* ^+^ *b* ^−^ *c* ^+^ therefore describes three tilts of different magnitudes. Tilts or rotations around *a* and *c* are in-phase (*a* ^+^, *c* ^+^) with respect to the layers above and below along the [100] and [001] axes, respectively, and tilting around *b* is in anti-phase (*b* ^−^) with respect to the layers along the [010] axis. Glazer’s assumptions initially identified 23 distinct tilted systems but Howard & Stokes (1998 ▸) reduced this to 15, showing that several were identical if crystallographic conventions such as origin choice and unique axis were taken into account. However, there are additional tilting scenarios when the octahedra are allowed to distort. Tilt has also been defined by Beanland (2011 ▸) in terms of centrosymmetric octahedra, but with a tensor description that defines the positions of the vertices of the octahedra. While this method avoids some of the limitations of Glazer’s method, it still relies on enforcing symmetry. Beanland’s method can accommodate grain boundary scenarios but requires an external frame of reference to define tilts. In the work of both Glazer and Beanland, tilt is still defined qualitatively; the magnitude of the tilts (except through Rietveld analysis of diffraction data) and their dynamic nature is not quantified.

Tilting can be observed experimentally either through refinement of the crystal structure data obtained by X-ray or neutron diffraction or more directly by analysis of the associated superlattice reflections in neutron, X-ray and electron diffraction patterns (Woodward & Reaney, 2005 ▸). In the case of electron diffraction, tilting may be investigated by considering the superlattice reflections along specific directions of the crystal or zone axes. By obtaining selected-area diffraction patterns (SADPs), the Glazer tilt system can be identified following the precepts of Woodward and Reaney. In brief, in-phase and anti-phase superlattice reflections generally give rise to ½{ooe} and ½{ooo} where *h* ≠ *k* and o = odd and e = even. Their appearance and distribution in equivalent major pseudo-cubic directions/zone axes (〈001〉, 〈110〉 and 〈111〉) can either define the tilt system or distinguish between likely configurations. These configurations can be determined in real time during examination of a perovskite on the transmission electron microscope so that further experiments (*e.g.* dark-field and high-resolution imaging) can be used to determine key crystallographic features that influence properties such as orientation and translational domain wall types and distributions.

SADPs are slices of reciprocal space passing through the origin and normal to the direction of interest (Fultz & Howe, 2001 ▸). In a cubic perovskite, superlattice reflections are not present provided that thermally induced oscillations of the ions can be ignored. Discrete long-range-order reflections in SADPs were formerly captured through short exposure (5–20 s) on film but researchers investigated diffuse scattering through longer exposure (50–100 s). On modern transmission electron microscopes, image intensifier and capture systems are routinely fitted and the sensitivity can be adjusted to record diffuse scattering in electron diffraction patterns which has its origin in weak/local structural correlations but whose intensity is enhanced through dynamical scattering as electrons travel through the sample.

Although diffraction techniques have revealed much about the tilting behaviour in perovskites, the standard interpretation (Beanland, 2011 ▸) assumes a homogeneous tilt pattern throughout the whole material and regular, undistorted octahedra. Refinement of neutron scattering data is often used to extract quantitative information about the O substructure but this requires beam time on central facilities. Atomistic simulations can give an accurate localized view of the atomic relaxations and demonstrate how defects, compositional variations and, ultimately, temperature can alter tilt angles. To achieve this, however, we must build a robust method for extracting the tilted phase from such simulations and relate this directly to the experimental interpretation. Codes exist (Wells & Sartbaeva, 2015 ▸) to separate the pure rotations of the octahedra from the distortions but these do not determine the tilting patterns. In this paper we define the degree of ‘tilt’ and the phase of the tilt using molecular dynamics simulations. We can then predict the diffraction data, in our case primarily SADPs, and so understand the dynamics and potentially diffuse scattering which result in the experimental observations.

## 2. Defining tilt when the octahedra are distorted

**Figure 3:**
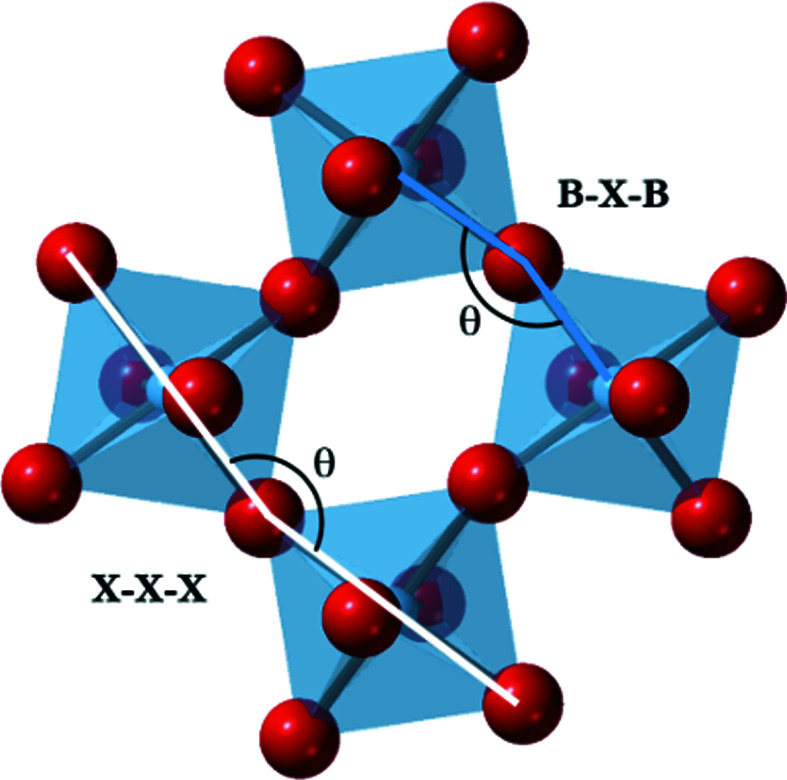
We define tilt angle as the angle between the connected vectors, [*XX*]_1_ and [*XX*]_2_, in an *ABX*_3_ perovskite. Vectors [*XX*]_1_, [*XX*]_2_ run from one corner of an octahedron to the opposite corner, crossing near the centroid of the octahedron. This is compared with the angle between vectors [*BX*]_3_ and [*XB*]_4_.

Within atomic scale simulations, distortions will naturally occur within the octahedra that break the centre of symmetry. It is then not sufficient to describe our structures using projections onto the pseudo-cubic planes, or to use deformation tensors. Instead, we define tilt using the angles between the vectors that meet at the shared corners of the octahedra (Fig. 3 ▸). In Fig. 3 ▸ the tilt is defined as the angle between the corner-sharing vectors, [*XX*]_1_ and [*XX*]_2_, where the vectors are between the *X* (red) ions at the opposite corners of the octahedra. We choose this definition, instead of using the vectors [*BX*]_1_ and [*XB*]_2_ (where the vectors are the corner-sharing vectors connecting the *B*-site ion to the shared corner ions) because the *B*-site ions are free to move off the centroid of the octahedra.

We then compute a distortion index, *D* [equation (2)], for the octahedra in our system,

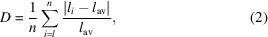

where *n* is the coordination number, *l_i_* is an individual cation–oxygen distance and *l*_av_ is the mean cation–oxygen distance in an octahedron. {Note that this differs slightly from the distortion index of Tillmanns *et al.* (1985 ▸) which is defined as Δ = 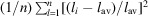 .} We also use the definition from Robinson *et al.* (1971 ▸) to compute the quadratic elongation, λ, and the variance of the bond angles, 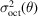 , of the octahedra,

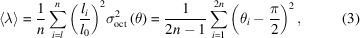

where, as before, *n* is the coordination number and *l_i_* is a cation–oxygen distance but *l*_0_ is the distance from the centre to the vertex of a perfect octahedron (*i.e.* with *O_h_* symmetry) whose volume is equal to that of the distorted octahedron. We also define the *B* site–*A* site ratio (*B*/*A* ratio) as the volume of the eight octahedra that surround an *A* site, divided by the volume of the *A* site. The volumes of the *B*-site octahedra and *A*-site cuboctahedra are obtained by splitting them into their constituent (irregular) tetrahedra and then using the Cayley–Menger determinant to obtain the volumes of all the tetrahedra. The volume of an irregular tetrahedron, *V*_tet_, is given by

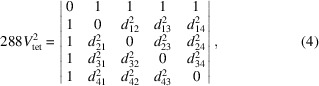

where *d_ij_* is the distance between vertex *i* and vertex *j* of the tetrahedron (Gritzmann & Klee, 1994 ▸). In an ideal case, where the sides of the octahedra and the sides of the cuboctahedra are the same (and so the system is cubic) the *B*/*A* ratio is equal to 1.6. A value greater than 1.6 implies *A*-site compression (or *B*-site stretching), and a value less than 1.6 means that the *B* sites are compressed (or the *A* sites have stretched). Our in-house program, *PALAMEDES*,^1^ performs this analysis of the structure of the octahedra and cuboctahedra for a molecular dynamics simulation of a perovskite, and computes the angles and phase of tilt for each octahedron at every timestep.

**Figure 4:**
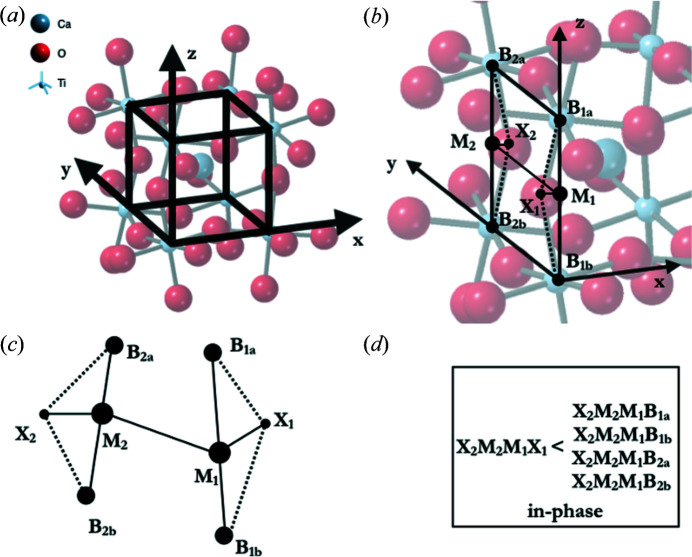
The definition of tilt within a perovskite, based upon the geometric connectivity of the atoms. In (*a*) we define the cube made by the *B*-site ions. In (*b*) we define two edges of the cube (*B*_1*b*_ *B*_1*a*_, *B*_2*b*_ *B*_2*a*_), their midpoints (*M*_1_, *M*_2_), and then vectors between these midpoints and both the *B* sites (*M*_1_ *B*_1*b*_, *M*_1_ *B*_1*a*_, *M*_2_ *B*_2*b*_, *M*_2_ *B*_2*a*_) and the *X* (*M*_1_ *X*_1_, *M*_2_ *X*_2_). In (*c*) the midpoints are connected by a vector (*M*_1_ *M*_2_), which allows us to define the ‘torsional’ angles (*X*_1_ *M*_1_ *M*_2_ *X*_2_, *X*_1_ *M*_1_ *M*_2_ *B*_2*a*_, *X*_1_ *M*_1_ *M*_2_ *B*_2*b*_, *B*_1*a*_ *M*_1_ *M*_2_ *X*_2_, *B*_1*b*_ *M*_1_ *M*_2_X_2_). We define the ‘torsional’ angle *X*_1_ *M*_1_ *M*_2_ *X*_2_ as follows. Looking in the direction *M*_1_ *M*_2_, the ‘torsional’ angle is the clockwise angle through which it is necessary to rotate the line *X*_1_ *M*_1_ such that the planes *X*_1_ *M*_1_ *M*_2_ and *M*_1_ *M*_2_ *X*_2_ are superimposed. The other ‘torsional’ angles are defined analogously. The system shows in-phase tilt if *X*_2_ *M*_2_ *M*_1_ *X*_1_ < min(*X*_1_ *M*_1_ *M*_2_ *B*_2*a*_, *X*_1_ *M*_1_ *M*_2_ *B*_2*b*_, *B*_1*a*_ *M*_1_ *M*_2_ *X*_2_, *B*_1*b*_ *M*_1_ *M*_2_ *X*_2_) as noted in (*d*).

The computation of tilt is done by comparing the edges of opposite faces of the distorted cube formed by the *B*-site ions about the *A* site [see Fig. 4 ▸(*a*) – this cube corresponds to the pseudo-cubic cell used in the experimental analysis]. For each edge, the [*BB*] vector is defined and the midpoint along this vector is determined. For each edge, the vector between this midpoint and the *X* ion that is shared by the two *B* ions that share this edge is found. Finally, the vector between the two midpoints is found. With this system defined, the phase of the tilt is simply found by finding the ‘torsional’ angles between the two *X* ions, and between each of the *X* ions and the *B*-site ions. [We use the standard construction (see *e.g.* Leach, 2003 ▸) to define the ‘torsional’ angle: see Fig. 4 ▸(*c*) and the caption for details.] If the angle between the two *X* ions is less than all other angles determined this way, the tilt is defined as anti-phase; otherwise, it is in-phase (see Fig. 4 ▸).

**Figure 5:**
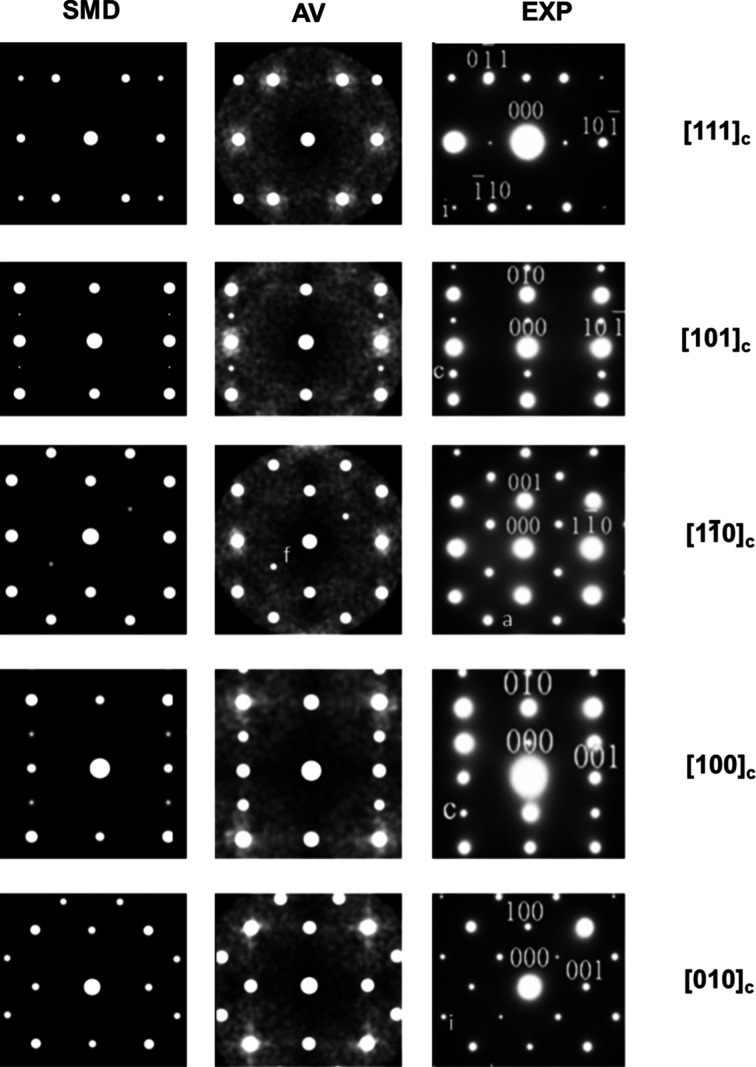
Simulated pattern from a single molecular dynamics (SMD) configuration, simulated pattern from 1000 averaged (AV) configurations (see Section 3) and experimental (EXP) selected-area patterns from CaTiO_3_ indexed in a pseudo-cubic setting. a = anti-phase, i = in-phase, c = cation shift and f = forbidden reflections (by symmetry and discounting double diffraction). Experimental data from Woodward & Reaney (2005 ▸).

With the tilt phase and tilt angles about an *A* site now defined, we can compute the average tilt phase for the entire simulation in each pseudo-cubic unit-cell direction. This is labelled SMD (single molecular dynamics) in Fig. 5 ▸. We score an in-phase tilt with +1, and an anti-phase tilt with −1 for each possible set of six atoms [as shown in Figs. 4 ▸(*b*) and 4 ▸(*c*)] in a simulation. The average is then constructed by summing the scores and dividing by the number of sets of atoms. If the average tilt of the entire simulation for one of the directions is close to +1 it is an in-phase tilt, if close to −1 it is an anti-phase tilt and if close to 0 the tilt is neither in-phase nor anti-phase. Such a zero could indicate a random ordering of tilt, an oscillation between the two configurations or perhaps a shift across the cell. However, we must also consider the average angle of tilt, 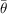 (where θ is defined in Fig. 3 ▸). If that angle is close to 180°, the system has no real tilt and the phase of the tilt is simply an artefact.

To ensure that we are not masking any short-range order, in addition to computing the average tilt phase for the SMD trajectory, we also compute an ‘average persistent tilt phase’, labelled AV in Fig. 5 ▸. To do this, we consider the lines of *A* sites along each pseudo-cubic unit-cell direction. For a given direction and a given *A* site, we define three groups of *A* sites of different lengths. These are: (i) the given *A* site and the neighbouring *A* sites (on both sides in the given direction); (ii) the given *A* site and the first two *A*-site neighbours (on both sides in the given direction); (iii) the given *A* site and the first three *A*-site neighbours (on both sides in the given direction).

We compare the tilt phases about each of the *A* sites within each group (where we are still calculating the tilt phase only between neighbouring *A* sites). If the tilt phase about all these *A* sites is anti-phase, a score of −1 is given. If the tilt phase about all of the *A* sites is in-phase, a score of +1 is given. For all other cases a score of 0 is given. We then calculate an average score for all the groups of a given size and direction. Comparing these average scores for the groups and directions gives information about short-range order in the tilting structure.

A pseudo-cubic frame of reference is used in the experimental analysis but the molecular dynamics simulations use supercells based on the orthorhombic structure. Simulation outputs have therefore been converted to the pseudo-cubic setting to compare simulations with experimental results. The relationship between the orthorhombic directions for a cell in which the *b* axis is doubled ( 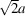 , 2*a*, 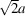 ) and the pseudo-cubic ( 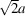 , 2*a*, 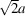 ) cell typically used when defining tilts is [001]_c_//[001]_o_, [100]_c_//[110]_o_, [010]_c_//[110]_o_ where subscripts c and o are cubic and orthorhombic, respectively. (This also gives the relations [110]_c_//[010]_o_, [101]_c_//[111]_o_ and [111]_c_//[021]_o_ which we shall require later.)

## 3. Simulation details

Molecular dynamics (MD) simulations of BaTiO_3_ and CaTiO_3_ were performed using the *DL_POLY 4* simulation package (Todorov *et al.*, 2006 ▸), for an NPT (N, number of particles; P, pressure; T, temperature) Nosé–Hoover ensemble (with relaxation times of 0.1 ps for both thermostat and barostat) using a timestep of 0.5 fs. For both systems, the simulation box size was approximately 100 × 100 × 100 Å, *i.e.* a 20 × 20 × 20 periodic array of orthorhombic unit cells containing 160 000 atoms. This size of simulation box approaches that of a small-aperture SADP sample. The simulation was run for 5000 timesteps to achieve equilibrium, and up to a further 10 000 timesteps used to produce the trajectory for analysis. For BaTiO_3_ (used as a comparison but diffraction data not shown) we used the published force fields of Freeman *et al.* (2011 ▸). The force field for CaTiO_3_ simulations uses the Ca–O interaction from Dawson *et al.* (2013 ▸) and a modified Ti–O interaction (see Tables 1 ▸ and 2 ▸). The force fields predict local environment effects for both Ba_1−*x*_Ca_*x*_TiO_3_ and Ba_1−*x*_Sr_*x*_TiO_3_ (Dawson *et al.*, 2014 ▸) which can be correlated with the Curie temperature. Similar effects have been seen in the reverse Monte Carlo refinements of a combination of neutron diffraction, X-ray absorption fine structure and diffuse electron scattering data of Levin *et al.* (2014 ▸) for the case of Ba_1−*x*_Sr_*x*_TiO_3_.

The use of simplified models is necessary because of the size and number of the calculations required. We therefore constructed rigid ion models by removing the shells from the shell model while retaining the full ionic charges. These models give similar bulk properties to the shell model except for the high-frequency dielectric constant. Although both types of model give stable phonons, the vibrational densities of states are (necessarily) different, particularly for the optic modes. The acoustic modes (particularly those projected onto the alkaline earth metal cation) are much more similar. See the supporting information for further details and comparison with a published vibrational density of states.

It is important to consider the details of how the simulated configurations are sampled in practice. Simulations typically calculate trajectories that last no more than a few nano­seconds. However, experimental techniques collect data over much longer periods (seconds to minutes). Experimental methods also sample the structure much less frequently than MD simulations. Typical values (Williams & Carter, 2009 ▸) of current (0.5 µA), diameter (10 nm) and accelerating potential (200 kV) for the beam give an electron velocity of 2.1 × 10^8^ ms^−1^ and a number density of electrons of 1.5 × 10^4^ m^−3^ (*i.e.* 1.2 × 10^−3^ electrons per nm length of the beam). Hence a transmission electron microscopy sample of thickness 10 nm exposed to such a beam will interact with an electron about once every 60 ps. This is about five orders of magnitude less frequent than the MD sampling (where the timestep is usually of the order of femtoseconds). Sampling the MD simulation every *n*th timestep (to correspond with the experiment), however, would not be appropriate as the motion of the atoms may involve regular periodic oscillations whose importance could be over-emphasized by a regular sampling process. With this in mind, we employ a Monte Carlo sampling of the MD trajectory where configurations are randomly selected from the whole trajectory. Based upon this sampling an averaged structure is generated and used to predict the SADP. It is important to realize that, although the position of each atom is the average position of the atoms in the sampled configurations, this does not mean that each atom is on the position expected from consideration of the perfect crystal structure. The simulation cell is large enough (8000 orthorhombic unit cells) to contain a thermal distribution of atomic positions for each perfect crystal structure position. The only enforced periodicity is at the level of the simulation cell, not the orthorhombic unit cell. This means that SADPs predicted using this cell can (and indeed do) show the presence of diffuse scattering.

## 4. Results

A total of 10 000 timesteps from the 50 K CaTiO_3_ simulation were sampled randomly, increasing the number of configurations, to obtain the data shown in Table 3 ▸. Most values converged quickly (including the standard deviation), suggesting that 100 samples would be enough. However, this rate of convergence depends on the system and the time and size of the oscillations. We therefore use a more conservative sampling. A total of 1000 random samples were used for the results shown in Fig. 5 ▸.

The sampled configurations were then used to calculate the tilt pattern with *PALAMEDES*. The code correctly identifies the phase tilt for CaTiO_3_ as two anti-phase and one in-phase tilt which corresponds to the *a* ^−^ *b* ^+^ *a* ^−^ (*Pbnm*) case in Glazer’s nomenclature. It also correctly identifies the lack of correlated tilt in BaTiO_3_, giving *a* ^0^ *a* ^0^ *a* ^0^ (Table 4 ▸). Examination of the actual angles recorded (Table 5 ▸) shows no effective tilt for the octahedra in BaTiO_3_ [the *X*—*X*—*X* angles (the tilt angle, θ, shown in Fig. 3 ▸) are very close to 180°]. In CaTiO_3_, the octahedra all tilt ∼20° in the *a*, *b* and *c* directions. This degree of tilt does not change significantly from 50 to 350 K but an increase in the standard deviation is observed due to ionic displacements becoming greater and more random as temperature increases. We note that the octahedral volume does not change and therefore the small volume increase seen at this temperature range relates to the fluctuation in the tilt causing a small expansion of the cell.

The *Single Crystal 4* code is used to generate SADPs for our simulated crystal systems and in Fig. 5 ▸ the output is compared with experimental patterns from Woodward & Reaney (2005 ▸). The weak scattering arises from local distortions that break long-range symmetry. They are kinematic in origin and in real patterns would be enhanced through dynamical scattering. All diffuse scattering has a kinematic origin, although it is often too weak to observe without multiple scattering events. The simulated diffraction patterns identify possible diffraction spots (given the kinematic approximation) including the required ½{ooe} and ½{ooo} superlattice reflections, conventionally associated with the *a* ^−^ *b* ^+^ *a* ^−^ tilt system distributed correctly within the major pseudo-cubic zone axes of interest ([001], [110] and [111]). The pattern can be generated from either the perfect CaTiO_3_ crystal (*i.e.* a single configuration selected from a MD run) or we can use a Monte Carlo sampled system which includes the natural thermal fluctuations of the system.

The Monte Carlo sampling could be viewed as giving an insight into potential weak diffuse scattering that can arise because of local correlations of tilting rather than the average tilt structure. Indeed, diffuse scattering and weak reflections do appear around the fundamental spots, particularly in [100] and [010] zones with extremely weak, diffuse intensities at ∼¼00 positions. Undoped CaTiO_3_ has never been reported to show such superlattice reflections but Howard *et al.* (2008 ▸) have reported a NaNbO_3_-like cell with such quadrupling in Sr-doped CaTiO_3_. Reaney *et al.* (2011 ▸) suggested that the NaNbO_3_-like structure could be stabilized through frustration of anti-phase and in-phase tilting close to a phase boundary between two different tilt systems. If the ¼00 intensities genuinely reflect local tilt correlations in CaTiO_3_, then the appearance of the NaNbO_3_-like structure in Sr-doped ceramics is less surprising as the simulations suggest that there are pre-existing local deviations in the CaTiO_3_ end member from the average that favour a compound quadrupled tilt axis (as seen in NaNbO_3_).

At this stage however, interpretation of diffuse scattering (kinematic contribution) is speculative, requiring significant further study which must also include an appreciation of multiple scattering as electrons pass through the sample. Forbidden (f) reflections according to the Glazer tilt system for CaTiO_3_ (*a* ^−^ *b* ^+^ *a* ^−^) do occur at ½ ½ ½ positions which in previous work have only ever been attributed to double diffraction from, *e.g.*, 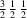 through 100 planes. These simulations would suggest that there is a kinematic intensity, albeit weak, in these positions which requires further investigation but may result from a lowering or breaking of local symmetry. For comparison, simulations of the ‘untilted’ structure of BaTiO_3_ were performed (not shown) which confirmed that no superlattice reflections are present, consistent with the *P*4*mm* symmetry at room temperature.

**Figure 6:**
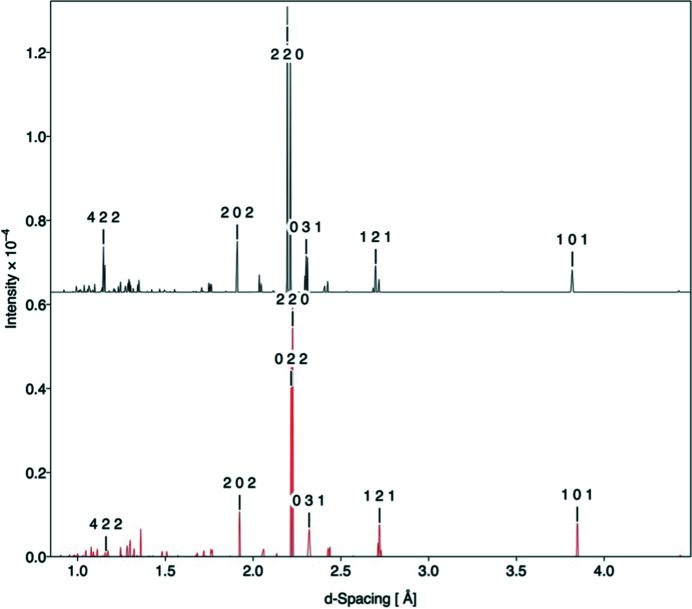
Time-of-flight neutron diffractogram for the CaTiO_3_ system at 50 K. Top: experimental pattern (using data from Knight, 2011 ▸). Bottom: simulated pattern obtained from molecular dynamics trajectories using *Crystal Diffract* from the *CrystalMaker* code suite (CrystalMaker, 2021*a* ▸).

To further illustrate the potential of this approach, a neutron diffraction pattern was generated using the sampled average structure (Fig. 6 ▸) which compares well with experiment, suggesting that the force field used reproduces CaTiO_3_ accurately. As an average structure was used, thermal effects of motion were included in the pattern calculation, removing the necessity of calculating thermal ellipsoids to represent thermal effects.

## 5. Related literature

The following references are cited in the supporting information: Beran *et al.* (1996 ▸), Cockayne & Burton (2000 ▸), Dawson *et al.* (2013 ▸), Lebedev (2009 ▸), Parlinksi *et al.* (2001 ▸), Souza & Rino (2011 ▸), Zhang *et al.* (2016 ▸).

## 6. Conclusions

We have demonstrated the ability of the *PALAMEDES* code to identify and characterize tilt patterns in perovskites rather than just providing tilting angles for the octahedra which the user must then subject to further analysis [as is the case for current codes such as *GASP* (Wells & Sartbaeva, 2015 ▸)]. We have also used the simulated trajectories to obtain time-averaged electron diffraction patterns of perovskites with the correct superlattice reflections and time-of-flight neutron spectra using standard codes such as *Single Crystal 4* (CrystalMaker, 2021*b* ▸) and *Crystal Diffract 6* (CrystalMaker, 2021*a* ▸). Our tilt analysis code, *PALAMEDES*, can readily distinguish between different tilt structures (as shown in the examples of BaTiO_3_ and CaTiO_3_) and also returns the dynamic behaviour of the tilt angle oscillations, including averages, minima and maxima, and correlates this to the tilt phase. This gives us a notation analogous to that of Glazer. Our analysis has revealed that, while the tilt phase of CaTiO_3_ does not change on average with increasing temperature, the oscillations of the tilts do increase in magnitude. BaTiO_3_, however, is a far more rigid structure. This methodology can be used in conjunction with simulations and experiments to predict the diffraction patterns for new materials and differentiate between these materials in terms of the dynamic nature of the tilts. We tentatively propose that AV simulations give new information with regards to short-range order whereas SMD is sufficient to model fundamental structure and tilt substructure.

## Supplementary Material

Comparison of the behaviour of the forcefields used with ab initio simulations. DOI: 10.1107/S2053273322011949/lu5021sup1.pdf

## Figures and Tables

**Table 1:** Ca—O interaction from Dawson *et al.* (2013 ▸) Buckingham potential 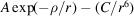 .

Ions		*A* (eV)	ρ (Å)	*C* (eV Å^6^)
Ca	O	1375.0	0.3325	15.21

**Table 2:** Modified Ti—O interaction Lennard-Jones 7–6 potential 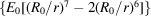 .

Ions		*E*_0_ (eV)	*R*_0_ (Å)
Ti	O	0.01194	4.719

**Table 3:** *PALAMEDES* output for volume and angles of octahedra in CaTiO_3_ using random sampling

No. of random samples	Mean octahedral volume (Å^3^)	Std deviation of volume	Min. volume (Å^3^)	Max. volume (Å^3^)	Mean angle *X*+ (°)	Min. angle *X*+ (°)	Max. angle *X*+ (°)	Std deviation of angle *X*+ (°)
100	9.919	0.064	9.369	10.188	159.09	154.00	164.61	0.976
200	9.919	0.064	9.337	10.197	159.08	154.00	164.85	0.977
500	9.919	0.064	9.372	10.213	159.08	154.02	164.83	0.978
1000	9.919	0.064	9.367	10.217	159.09	154.12	164.72	0.977
2000	9.919	0.064	9.357	10.218	159.08	154.00	165.03	0.977
5000	9.919	0.064	9.348	10.214	159.08	153.98	165.03	0.977

**Table 4:** *PALAMEDES* tilt phase output for CaTiO_3_ and BaTiO_3_

	Temperature (K)	*a*	*b*	*c*
CaTiO_3_	50	−1	1	−1
CaTiO_3_	150	−1	1	−1
CaTiO_3_	350	−1	1	−1
BaTiO_3_	350	0	0	0

**Table 5:** *PALAMEDES* angles output for randomly sampled CaTiO_3_ and BaTiO_3_

	Octahedral volume (Å^3^)		*X*+ (°)		*Y*+ (°)		*Z*+ (°)	
Temperature (K)	Mean	Std deviation	Mean	Std deviation	Mean	Std deviation	Mean	Std deviation
CaTiO_3_-50	9.93	0.06	159.03	0.80	159.03	0.80	158.65	0.86
CaTiO_3_-150	9.93	0.13	159.56	1.56	159.28	1.44	158.92	1.52
CaTiO_3_-350	9.93	0.12	160.17	2.74	160.17	2.74	159.82	2.73
BaTiO_3_-350	10.65	0.02	179.8	0.04	179.7	0.06	179.8	0.04

## Footnotes

1 See Virgil *Aeneid* Book 2 lines 81–85; Pausanias *Description of Greece*, Book 2, Section 20.3. The program is currently written in Fortran but we hope to convert it to Python for easier distribution. A copy of the Fortran source is available on application to the authors.
