# Supplementary material for: Dynamic tilting in perovskites
Source: Acta Crystallogr A Found Adv. 2023 Jan 23;79(Pt 2):163–70. doi: 10.1107/S2053273322011949 (PMC9979940; doi:10.1107/S2053273322011949)
Supplement: Supplementary file 1 [file a-79-00163-sup1.pdf]

FOUNDATIONS ADVANCES

Volume 79 (2023)

Supporting information for article:

Dynamic Tilting In Perovskites

Christopher Handley, Robyn Ward, Colin Freeman, Ian Reaney, Derek Sinclair and John Harding

Dynamic Tilting In Perovskites: Supplementary information

C. M. Handley[a], R. E. Ward,[a,b] C. L. Freeman[a], I. M. Reaney[a], D. C. Sinclair[a] and J. H. Harding[a*]

[a] Department of Materials Science and Engineering, University of Sheffield, Sir Robert Hadfield Building, Mappin Street, Sheffield, S1 3JD.

[b] Digital Research Service, University of Nottingham, University Park, Nottingham NG7 2RD.

1. Forcefield for $CaTiO_3$

The forcefield for $CaTiO_3$ is that of Dawkins *et al* (2013) with minor modifications to the Ti - O interaction. Because of the length and number of the molecular dynamics runs, we have converted the shell model to a rigid ion model by removing the shells but retained the full ionic charges on the ions. The effects of this on the crystal structure are shown in Table S1.

	a (Å)	b (Å)	c (Å)	*u*	*v*	*t*	*w*	*x*	*y*	*z*
Experiment (Beran,1996)	5.440	7.639	5.380	0.4644	0.0065	0.5161	0.5711	0.2112	0.0373	0.2897
Shell model	5.4401	7.6788	5.4106	0.4839	0.0022	0.5161	0.5704	0.2153	0.0336	0.2852
Rigid ion model	5.4401	7.6788	5.4106	0.4865	0.0020	0.5149	0.5642	0.2167	0.0328	0.2837
DFT (Parlinski, 2001)	5.3596	7.4020	5.1724	0.4699	0.0988	0.5142	0.5593	0.1982	0.0517	0.2994

Table S1: Comparison of rigid ion model and shell model with experimental data and density functional (DFT) calculations (all referred to the *Pnma* space group. Coordinates are Ti (0,0,0); Ca(*u,1/4,v*); O(*t, 1/4, w*), O(*x, y, z*)).

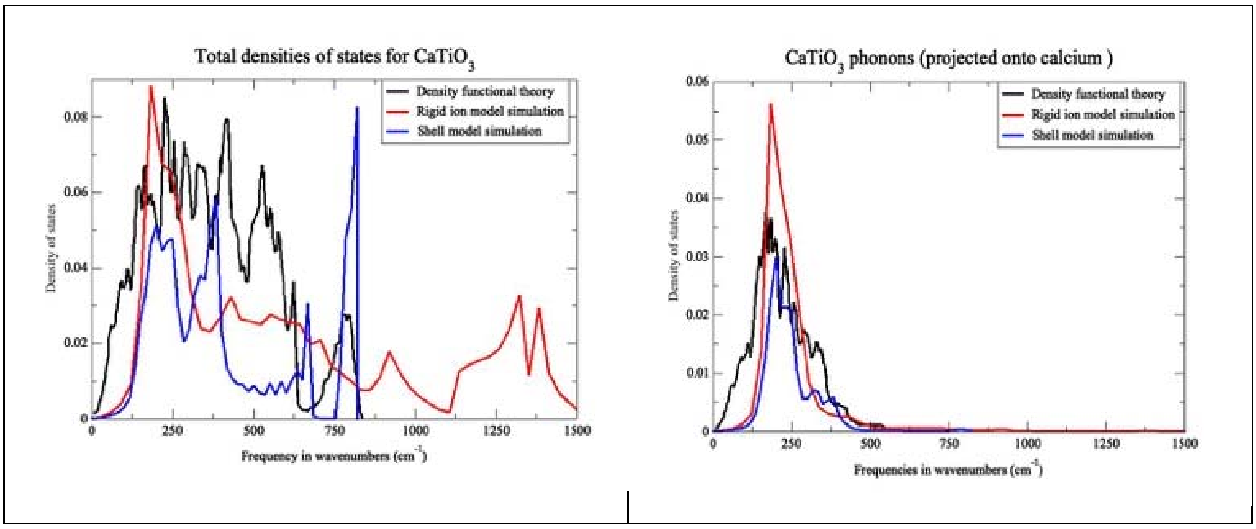

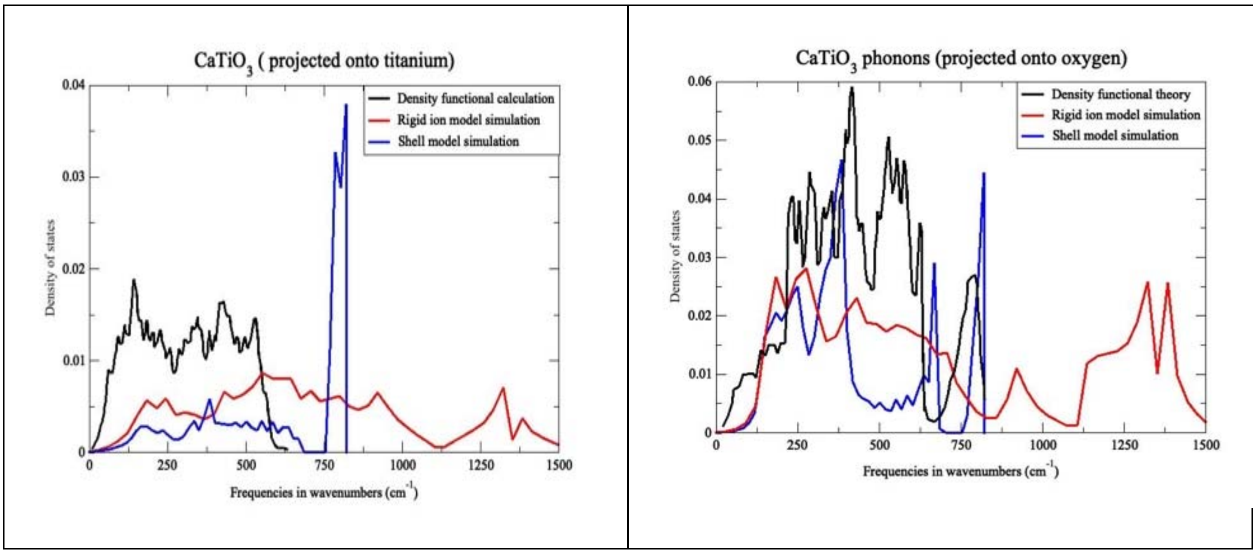

Figure S1: Phonon density of states for $CaTiO_3$. Comparison of density functional theory (Parlinski *et al* 1991) with a rigid ion model and shell model with the same interatomic interactions but a shell on the oxygen ions (shell charge, Y = -2.45|*e*|, spring constant, *k* = 25,0 eV Â$^{-2}$)

Both Parlinski *et al* (2001) and Cockayne & Burton (2000) present calculated values of the dispersion curves for orthorhombic $CaTiO_3$. Others (e.g. Lebedev (2009)) give results for the cubic form (including imaginary modes). These curves are too complex to permit a meaningful comparison but Parlinski also gives results for the calculated density of states. The differences between the rigid ion and shell models are mainly due to the failure of the former to include electronic polarisation effects. The Lyddane-Sachs-Teller relationship states that

$$\left(\frac{\omega_L}{\omega_T}\right)^2 = \frac{\varepsilon_0}{\varepsilon_\infty} \qquad (1)$$

where ω_L, ω_T are the longitudinal optic (LO) and transverse optic (TO) modes at the Γ point and ε_0, ε_∞ are the static and high frequency dielectric constants respectively. Since for the rigid ion model $\varepsilon_\infty = 1$ it is inevitable that the LO - TO gap will be overestimated. As here, the LO mode is usually the most affected, being much too high in frequency. However, the low frequency modes are in much better agreement with each other and with the density functional calculations, particularly those which are dominated by the behaviour of the alkaline earth cation. These are most likely to be important in the dynamic tilting behaviour. The only other reported phonon density of states obtained using a rigid ion model (to our knowledge) is that of Souza & Rino (2011). This performs rather better than our rigid ion model since it uses partial charges but this makes it useless for performing calculations of defects (particularly those involving aliovalent dopants). However, as can be seen from Figure 13 of Souza & Rino, at a qualitative level the strengths and weaknesses of the two models are similar.

Similar behaviour can be seen in the $BaTiO_3$ phonon density of states as shown in Figure S2. The behaviour of the phonons projected onto the barium ion appear to be broadly similar to that shown in Figure 2 of Zhang *et al* (2016) but a direct comparison is not possible since it is unclear how the densities of states reported in Zhang *et al* have been normalised.

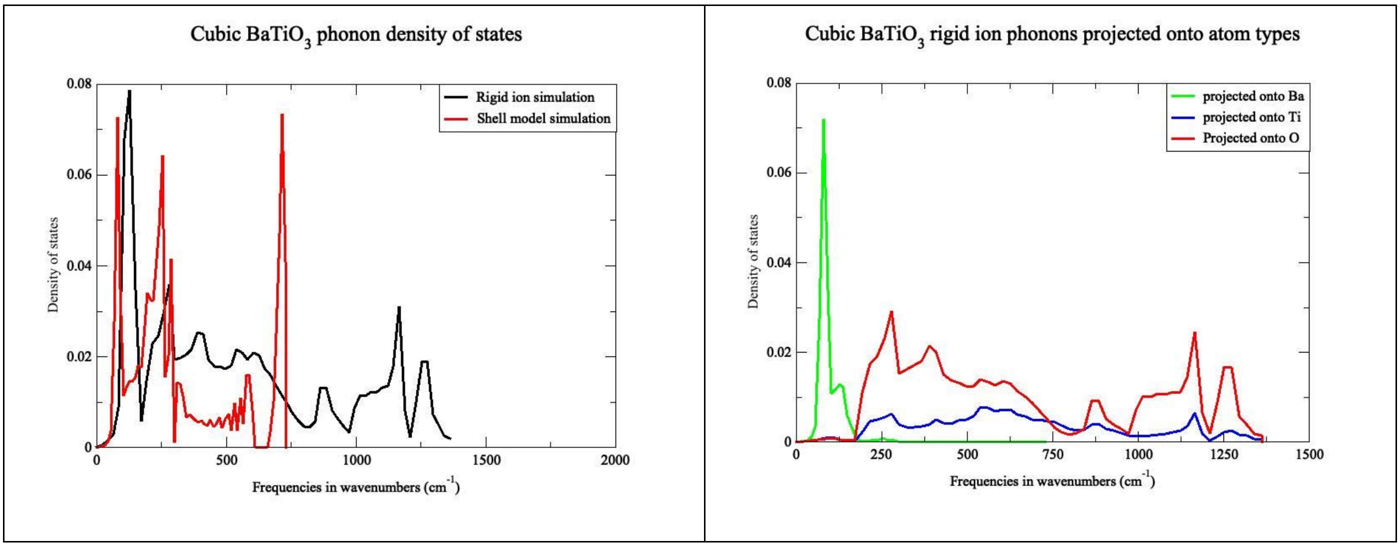

Figure S2 Comparison of phonon density of states for $BaTiO_3$. **Left**: Rigid ion and shell models. **Right:** Projection onto the atom types for the rigid ion model.

References

Beran, A., Libowitzky, E. & Armbruster T., (1996) *Canad. Min.* **34,** 803-809.

Cockayne, E. & Burton B. P., (2000) *Phys. Rev. B***62,** 3735-3743.

Dawson, J. A., Li, X., Freeman, C. L., Harding, J. H. & Sinclair, D. C., (2013) *J. Mater. Chem. C***1**, 1574-1582.

Lebedev, A. I., (2009) *Phys. Solid State* **51,** 341-350.

Parlinski, K., Kawazoe, Y. & Waseda, Y., (2001) *J. Chem. Phys.* **114,** 2395-2400.

Souza, J. A. & Rino. J. P., (2011) *Acta Mater.* **59,** 1409-1423.

Zhang, H. Y., Zeng, Z. Y., Zhao, Y-Q., Liu, Q. & Cheng Y. (2016), *Z, Naturforsch.* **71**, 759-768.
